# Transcriptome Analysis of *ppdnmt2* and Identification of Superoxide Dismutase as a Novel Interactor of DNMT2 in the Moss *Physcomitrella patens*

**DOI:** 10.3389/fpls.2020.01185

**Published:** 2020-08-05

**Authors:** Darshika Singh, Radha Yadav, Shubham Kaushik, Nikita Wadhwa, Sanjay Kapoor, Meenu Kapoor

**Affiliations:** ^1^ University School of Biotechnology, Guru Gobind Singh Indraprastha University, New Delhi, India; ^2^ Vproteomics, Valerian Chem Private Limited Green Park Mains, New Delhi, India; ^3^ Interdisciplinary Centre for Plant Genomics and Department of Plant Molecular Biology, University of Delhi South Campus, New Delhi, India

**Keywords:** DNMT2, *Physcomitrella*, transcriptome, superoxide dismutase, stress

## Abstract

*DNMT2* is a DNA/tRNA cytosine methyltransferase that is highly conserved in structure and function in eukaryotes. In plants however, limited information is available on the function of this methyltransferase. We have previously reported that in the moss *Physcomitrella patens*, *DNMT2* plays a crucial role in stress recovery and tRNA^Asp^ transcription/stability under salt stress. To further investigate the role of *PpDNMT2* at genome level, in this study we have performed RNA sequencing of *ppdnmt2*. Transcriptome analysis reveals a number of genes and pathways to function differentially and suggests a close link between *PpDNMT2* function and osmotic and ionic stress tolerance. We propose *PpDNMT2* to play a pivotal role in regulating salt tolerance by affecting molecular networks involved in stress perception and signal transduction that underlie maintenance of ion homeostasis in cells. We also examined interactome of PpDNMT2 using affinity purification (AP) coupled to mass spectrometry (AP-MS). Quantitative proteomic analysis reveals several chloroplast proteins involved in light reactions and carbon assimilation and proteins involved in stress response and some not implicated in stress to co-immunoprecipitate with PpDNMT2. Comparison between transcriptome and interactome datasets has revealed novel association between *PpDNMT2* activity and the antioxidant enzyme Superoxide dismutase (SOD), protein turnover mediated by the Ubiquitin-proteasome system and epigenetic gene regulation. PpDNMT2 possibly exists in complex with CuZn-SODs *in vivo* and the two proteins also directly interact in the yeast nucleus as observed by yeast two-hybrid assay. Taken together, the work presented in this study sheds light on diverse roles of *PpDNMT2* in maintaining molecular and physiological homeostasis in *P. patens*. This is a first report describing transcriptome and interactome of DNMT2 in any land plant.

## Introduction

Methylation of cytosine residues (m^5^C) in transfer RNAs (tRNAs) is a conserved modification found in this class of non-coding RNAs across eukaryotes (Helm, 2006; Motorin et al., 2009). It has been described in the nuclear tRNAs in lower and higher eukaryotes including the single-celled green algae and the multicellular flowering plants (Wilkinson et al., 1995; Okano et al., 1998; Yoder and Bestor, 1998; Goll et al., 2006; Burgess et al., 2015). Methylation of tRNAs is known to stabilize RNA secondary structures and prevent its cleavage by ribonucleases under a variety of stress conditions and also affect developmental processes in both plants and animals (Rai et al., 2007; Schaefer et al., 2010; Tuorto et al., 2012; Burgess et al., 2015; David et al., 2017). There are two classes of RNA methyltransferases (RMTases) that catalyze m^5^C in tRNAs: the Transfer RNA specific methyltransferase 4 (TRM4) or NOP2/Sun domain protein 2 (NSUN2) in yeast and animals that has two paralogs in Arabidopsis (TRM4a and TRM4b) (Pavlopoulou and Kossida, 2009; Chen et al., 2010; Tuorto et al., 2012; Burgess et al., 2015; Motorin et al., 2016) and the Transfer RNA aspartic acid methyltransferase 1 (TRDMT1) or DNA methyltransferase 2 (DNMT2) (Goll et al., 2006). In Arabidopsis, both the RMTase catalyze m^5^C in the variable and anticodon loop in a number of tRNAs (Burgess et al., 2015; David et al., 2017).

DNMT2/TRDMT1 is a highly conserved cytosine methyltransferase in eukaryotes. It differs from other proteins in this family in lacking the N-terminal regulatory domains, being shorter in length and in methylating cytosines in both DNA and tRNAs (Wilkinson et al., 1995; Goll et al., 2006). It was first identified in the fission yeast, *Schizosaccharomyces pombe* as *pombe methyltransferase 1* (*pmt1*) and is known to be the only methyltransferase in the genomes of many model organisms including *Drosophila melanogaster*, *Dictyostelium discoidium*, and *Entamoeba histolytica* (Wilkinson et al., 1995; Okano et al., 1998; Yoder and Bestor, 1998). *DNMT2* expression is developmentally regulated in these organisms (Hung et al., 1999; Kuhlmann et al., 2005; Rai et al., 2007; Müller et al., 2013). Loss-of-function mutations in *DNMT2* does not have deleterious consequences on genomic methylation patterns, embryonic development, vegetative growth, chromosome stability, and mating type switching in mice, Drosophila, and yeast under standard conditions, but its role in affecting viability, silencing of mobile elements, innate immune response, telomere integrity, and histone H4K20 trimethylation is evident under stress conditions (Wilkinson et al., 1995; Lin et al., 2005; Katoh et al., 2006; Schaefer et al., 2010; Becker et al., 2012). DNMT2 also affects non-random segregation of sister chromatids in the asymmetrically dividing Drosophila male germline stem cells (Yadlapalli and Yamashita, 2013).

Though DNMT2 bears close similarity in sequence and structure to DNA methyltransferases, it however has weak DNA methyltransferase activity and a low preference for cytosines located as CpG in comparison to Cp(A/T) or CC(A/T)GG (Pinarbasi et al., 1996; Hermann et al., 2003; Kunert et al., 2003; Liu et al., 2003; Tang et al., 2003; Fisher et al., 2004; Mund et al., 2004; Kuhlmann et al., 2005). Active methylation of cytosines in tRNA molecules has been reported as a widely conserved function of DNMT2 in different organisms. DNMT2-mediated tRNA methylation is known to protect tRNAs against ribonucleases under heat and oxidative stress in Drosophila (Schaefer et al., 2010). This has also been correlated with small RNA homeostasis in flies (Durdevic et al., 2013).

Since DNMT2 has affinity for both DNA and RNA substrates and is involved in diverse biological processes, it is predicted to interact with a number of proteins. The putative interactors of DNMT2 based on high evolutionary rate covariation values have been identified as proteins belonging to either of the following categories: chromatin remodeling, transcription factor, gene expression regulation, DNA replication, stress response, and RNA editing (Vieira et al., 2018). The first *in vivo* protein partner reported for DNMT2 was the glycolytic protein Enolase that catalyzes conversion of 2-Phosphoglycerate to Phosphoenolpyruvate during glycolysis in Entamoeba (Tovy et al., 2010). Enolase binds to the *Entamoeba histolytica* 5-cytosine DNA methyltransferase (Ehmeth) and inhibits its tRNA methylation activity under glucose starvation conditions. In humans, DNMT2 interacts with a number of proteins involved in RNA processing, transcriptional control, and mRNA export (Thiagarajan et al., 2011). In plants such as Arabidopsis, interaction of AtDNMT2 with plant-specific histone deacetylases2 (HD2) proteins HD2A, 2B, and 2C suggests a close link between DNMT2 and epigenetic regulatory mechanisms (Song et al., 2010).

We have previously reported that the moss *Physcomitrella patens* encodes the longest eukaryotic DNMT2 with 477 residues that shares more than 50% identity with Arabidopsis DNMT2 and around 40% with corresponding proteins in yeast, humans, and mice, respectively (Malik et al., 2012; Arya et al., 2016). Although *PpDNMT2* differentially expresses in protonema and the leafy gametophores, its loss-of-function does not affect growth and development at these stages (Malik et al., 2012; Arya et al., 2016). Instead, *ppdnmt2* mutants display high sensitivity towards elevated salt and mannitol in the growth medium and are unable to recover from stress despite accumulation of normal levels of *PpDHNA* (encoding dehydrin) and the small heat shock protein encoding *PpHSP16.4* transcripts known to be essential for stress recovery in *P. patens* (Saavedra et al., 2006; Ruibal et al., 2012; Arya et al., 2016). To further investigate the functional capabilities of *PpDNMT2*, in this study we have analyzed the transcriptome of *ppdnmt2* by RNA sequencing and identified a number of differentially expressing genes and pathways that are affected by the loss of *PpDNMT2* function. Further, to gain insight into proteins existing in complex with PpDNMT2 *in vivo* we performed immunoprecipitation and identified proteins co-immunoprecipitated with PpDNMT2 by Liquid Chromatography-Mass Spectrometry (LC-MS). The transcriptome and interactome datasets reveal close link between *PpDNMT2* function and the antioxidant enzymes such as the Superoxide dismutases (SOD), osmotic and ionic homeostasis maintenance, regulation of protein turnover, and epigenetic regulation in *P. patens*.

## Materials and Methods

### Plant Material and RNA Isolation

For RNA sequencing, *Physcomitrella patens* Gransden (Wild type and *ppdnmt2*#8) was propagated on solid BCDAT medium (http://moss.nibb.ac.jp/protocol.html) overlaid with cellophane under continuous white light (40 µmol/m^2^/s) at 22–24°C. The cellophanes were shifted on fresh BCDAT medium every 7^th^ day, and tissue was harvested on the 24^th^ day. Total RNA was isolated from three biological replicates of each sample type using Trizol (Sigma) and cleaned up using GeneJET RNA clean-up and concentration micro kit (Thermo Scientific, Inc.) before eluting in RNase-free water. RNA quality and quantity were checked by fractionating samples on 1.2% agarose gel and using Nanodrop (Nanodrop LITE Thermoscientific).

### RNAseq Library Construction and Sequencing

RNA sequencing library was prepared using the Illumina-compatible NEBNext^®^ Ultra™ Directional RNA Library Prep Kit (New England BioLabs, USA) at Genotypic Technology Pvt. Ltd., Bengaluru, India following manual instructions. Briefly, 1 µg of total RNA was taken for mRNA isolation, fragmentation, and priming. The fragmented and primed mRNAs were then subjected to first strand synthesis in the presence of Actinomycin D (Gibco, Life Technologies, USA) followed by second strand synthesis. The double stranded cDNA was purified using HighPrep magnetic beads (Magbio Genomics Inc, USA) followed by end repair, adenylation and ligation to Illumina multiplex barcode adapters as per NEBNext^®^ Ultra™ Directional RNA library preparation kit protocol. Adapter ligated cDNAs were purified using HighPrep beads and subjected to 12 cycles of indexing-PCR (37°C for 15 min followed by denaturation at 98°C for 30s) cycling (98°C for 10 s, 65°C for 75 s) and at 65°C for 5 min to enrich adapter-ligated cDNA fragments. Final PCR products (sequencing library) were purified using the HighPrep beads followed by library quality control check. Illumina-compatible sequencing library was quantified using Qubit fluorometer (Thermo Fisher Scientific, USA), and its fragment size distribution (80 bp to 680 bp) was analyzed on Agilent 2200 Tapestation. Paired End (PE) sequencing (150 × 2) was then performed using the high-throughput Illumina HiSeq sequencing platform, HiSeq X Ten (Illumina, Inc.). Raw reads generated have been deposited in the NCBI BioSample database and can be accessed through Sequence Read Archive (SRA) accession # **SRR8555685, SRR8555689, SRR8555687, SRR8555688, SRR8555690, and SRR8574017**.

### Processing of Reads, Alignment Against *P. patens* Reference Genome and Differential Gene Expression Analysis

Quality of raw reads was checked using Fast QC (http://www.bioinformatics.babraham.ac.uk/projects/fastqc/), and these were pre-processed to remove adaptor sequences and low quality bases using Cutadapt (Martin, 2011). The processed reads were then aligned to the reference genome of *Physcomitrella patens_318_V3.3.* (https://phytozome.jgi.doe.gov/pz/portal.html) using TopHat-2.0.13 (Trapnell et al., 2009). The resulting files in bam format were used for downstream analysis. Transcript assembly, read count and differential gene expression was performed using Cufflinks-2.2.1 (Trapnell et al., 2010). Group wise comparison was performed to identify differentially regulated genes between wild type and *ppdnmt2#8* using cuffdiff. Up and downregulated genes were identified using log2 fold cut-off (+/–1) with P-value <= 0.05. Heat map was generated in R using Bioconductor package gplots.

### Gene Ontology and Pathway Analysis

All transcripts and proteins were annotated against Viridiplantae sequences downloaded from uniprot (http://www.uniprot.org/) and using The *Physcomitrella patens* Gene Model Lookup Database (PpGML DB; https://peatmoss.online.uni-marburg.de/ppatens_db/contact.php; Fernandez-Pozo et al., 2019), and GO terms were assigned accordingly. Fischer’s one-tailed test was used to calculate p-values. GO terms with p-values below the threshold of <=0.05 were considered statistically significant. GO enrichment analysis along with statistical filtering using Bonferroni correction was performed using PANTHER database (http://pantherdb.org/). Pathway analysis was performed using the KEGG pathways database (https://www.genome.jp/kegg/pathway.html).

### Antibody Preparation

Anti-PpDNMT2 antibody was synthesized using PpDNMT2-specific peptide 302-APPLLRKLIGDHYES-316 as antigen at BIONEEDS, Karnataka, India. For antisera development, rabbits were first immunized with the peptide antigen conjugated to a carrier protein followed by 2–4 rounds of boosters every 21 days. Titer was checked from 2^nd^ booster onwards, 10 days after injection, and antisera was collected after the desired titer was achieved. The sera were then affinity purified using Protein A.

### Stress Treatment, Protein Extraction and Western Blot

For AP-MS analysis, wild type protonema was propagated for nine days on solid BCDAT media under continuous white light at 22–25°C. The tissue was then transferred to liquid BCDAT for 5 days before supplementing the media with 400 mM NaCl and incubating further for 24 h. Tissue propagated under similar conditions and for the same period of time but without NaCl supplement was used as control. Three independent biological samples were used for control and salt-treated samples. Total protein was isolated by manually grinding 200 mg tissue in liquid nitrogen and adding 400 µl lysis buffer (150 mM NaCl, 10 mM Tris-HCl (pH 7.4), 1 mM EDTA, 1 mM EGTA (pH 8.0), 0.2 mM sodium ortho-vanadate, 0.2 mM PMSF, 1% Triton X-100 (Sigma), 0.5% NP-40 (Himedia) and 1× ProteaseArrest™ (G Biosciences) to the homogenate. After incubating the protein extract on ice for 30 min it was sonicated four times for 8 s each with at least an 8 s pause in between. Cell debris was then pelleted by centrifugation at 14,000 rpm for 10 min at 4°C. The clear supernatant was collected, and protein concentration was estimated by Bradford method using CB protein assay™ reagent (G Biosciences). *PpDNMT2* expression was confirmed by performing western blot. For this, 50 µg total protein was denatured by adding 1× SDS gel loading buffer (375 mM 1M Tris-Cl pH 6.8, 9% SDS, 50% Glycerol, 0.03% Bromophenol blue) and heated at 99°C for 10 min. The samples were fractionated using 12% SDS polyacrylamide gel at 70 V. After electrophoresis the gel was rinsed with sterile water and equilibrated in transfer buffer (25 mM Tris base, 192 mM Glycine, and 10% methanol) before blotting onto activated PVDF membrane (Merck Millipore) overnight at 4°C. After transfer, the membrane was washed once with TBS (20 mM Tris, 150 mM NaCl) and immersed in blocking solution (5% skimmed milk (Difco) in TBST: 1× TBS + 0.1% Tween 20) for 2 h at room temperature with constant shaking. Primary antibody (anti-PpDNMT2) at 1:500 dilution was then added and incubated for 60 min. After washing with TBST (15 min × 3), secondary antibody (Goat Anti-Rabbit IgG, HRP-conjugate, Merck Millipore) at 1:5,000 dilution was added, and membrane was further incubated for 60 min. It was then washed with TBST (15 min × 3) and developed using TMB (3,3′,5,5′-Tetramethylbenzidine, Sigma) substrate.

### Immunoprecipitation and Silver Staining

Endogenous PpDNMT2 was immunoprecipitated using anti-PpDNMT2 antibody (Ab, Ligand) coupled to Dynabeads-Protein A conjugate. Protein extracts (500 µg each) prepared from control, and treated samples were incubated with 7 µg anti-PpDNMT2 Ab overnight at 4°C with constant shaking. 50 µl Dynabeads-Protein A conjugate (Invitrogen, Thermo Fisher Scientific) was then added and incubated further for 2 h at 4°C. The tube was then placed on the magnetic stand to collect the Dynabeads-Protein A-Ab complex at the tube wall while the supernatant was discarded. The beads were washed twice with 500 µl lysis buffer. Three independent immunoprecipitation experiments each for control and salt-treated samples were carried out. For analyzing the bound antigen on SDS-PAGE, 2× SDS-PAGE sample loading buffer (G Biosciences) was added to the bead bound complex and boiled at 70°C for 10 min. After electrophoresis using 12% SDS-polyacrylamide gel silver staining was performed. For this, the gel was first washed with sterile water and then fixed for 60 min using 30% ethanol, 10% acetic acid adding fresh fixing solution after 30 min. After rinsing the gel with 20% ethanol (10 min × 2) and then sterile water for the same time, it was soaked for 1 min in 0.02% Sodium thiosulphate pentahydrate. After again rinsing with water (1 min × 2), the gel was impregnated with 12 mM silver nitrate for 30 min in dark without shaking. The gel was then briefly washed with water and developed using freshly prepared 3% potassium carbonate containing 10% sodium thiosulphate and 37% formalin for 45 min. The reaction was terminated using stop solution (0.3 M Tris and 2% acetic acid).

### LC-MS Analysis

After immunoprecipitation, the antigen along with the co-immunoprecipitated proteins was separated from Dynabeads-Protein A-Ab complex by adding 30 µl elution buffer (50 mM Glycine, pH 2.8). The pH of the eluate was adjusted using 1M Tris pH 7.5, and samples were sent to Central Instrumentation Facility at University of Delhi South Campus for LC-MS. Briefly, 25 µg of each protein sample was first reduced using 10 mM Dithiothreitol (DTT, Pierce) in 50 mM Ammonium BiCarbonate (ABC, pH~8) at 37°C for 30 min and then alkylated using 50 mM Iodoacetamide (IAA, Sigma) in 50 mM ABC (pH~8) in dark for another 30 min. After diluting the samples 10 times with water, the proteins were digested into smaller peptides using the serine protease Trypsin (Promega). This was added in the ratio of 1:50 (Trypsin : Lysate ratio) and incubated overnight at 37°C. The reaction was stopped by adding 10% Trifluoroacetic acid (TFA). Digests were then cleaned up using Pierce™ C18 Spin Columns according to manufacturer’s protocol and dried using speed vac (Thermo Savant DNA 120). The pellet was finally resuspended in Buffer-A (0.1% formic acid in water). The clarified peptide digested samples (1 µg each) were then resolved on a 50-cm long EASY-Spray column (50 cm * 75 um) PepMap RSLC filled with 2 µm-C18 resin on nano1200 chromatography system (ThermoFischer Scientific) attached to QExactive mass spectrometer equipped with nano-electrospray ion source. The peptides were loaded with Buffer A and eluted with a 5–40% gradient of Buffer-B (100% acetonitrile, 0.1% formic acid) for 102 min, 40–90% gradient for 1 min, followed by 90% gradient for 15 min at a flow rate of 300 nl/min with a total run time of 123 min.

The QExactive spray voltage was set at 4 kV, S lens RF level at 60 and ITC heated capillary temperature at 300°C. The MS data were acquired in positive polarity using a data-dependent method choosing the 10 most intense peaks with charge state +2 to +5, exclude isotope option enabled and dynamic exclusion time of 12 s. The MS1 (mass range 350–2,000 m/z) and MS2 scans were acquired in Orbitrap Mass analyser with resolution of 70,000 and 17,500 at m/z 200–2,000, respectively with polydimethylcyclosiloxane (PCM) ions (m/z = 445.120025), and lock mass option was enabled for internal recalibration during the run.

The MS1 or Full scan target was 3 × 10^6^ with a maximum fill time of 60 ms with mass range set to 350−2,000. Target value for MS2 or fragment scans was set at 1 × 10^5^, and intensity threshold was set at 8.3 × 10^2^. Isolation window of parent ion of interest was set at 1.5 m/z. Normalized collision energy for Higher-energy collisional dissociation (HCD) was set at 27. Peptide match option was set to preferred mode along with activation of isotope exclusion option.

### Protein Identification and Quantification

The RAW files containing the MS/MS spectra were analyzed using Proteome Discoverer (v2.2) software. The search engines Sequest HT and MS Amanda 2.0 based algorithms SEQUEST (Yates et al., 1995) and AMANDA (Dorfer et al., 2014) respectively, were used for matching peptides with proteins downloaded from Uniprot Proteins database for *Physcomitrella patens* containing 61,529 entries (https://www.uniprot.org/uniprot/?query=physcomitrella+organism%3Apatns&sort=score ). The protease Trypsin was used to generate peptides, and specificity for Trypsin/P was set (cleavage at the C terminus of “K/R: unless followed by “P”) with maximum missed cleavage value of 2. Carbamidomethyl on cysteine was considered as fixed modification, while oxidation of methionine and N-terminal acetylation were considered as variable modifications. For Sequest HT and MS Amanda 2.0 search, the precursor and fragment mass tolerances were set at 10 ppm and 0.5 Da, respectively. Minimum peptide length for search was set at 6, while maximum peptide length was set at 150. Both peptide spectrum match and protein False Discovery Rate (FDR) were set to 0.01 and determined using percolator node. Relative protein quantification was performed using Minora feature detector node of Proteome Discoverer 2.2 including a Minimum Trace Length of 5, Max. ΔRT of Isotope Pattern 0.2 min and considering only those with high PSM (peptide spectrum matches) confidence.

### Differential Analysis

Differential analysis was performed using protein abundance values in each sample type. The abundance values in each biological replicates were Log2 transformed followed by Z-score standardization for normalization of data. Statistical significance was inferred by performing Student t-test and proteins with p-value <= 0.05 and fold change **>=** 2 were considered statistically significant. Data was visualized using in-house R scripts.

### Construct Preparation

Constructs were prepared for yeast two-hybrid assays. PpDNMT2 coding region (1,434 bp) was amplified using the primer pair PpDNMT2TOPOY2HFP and PpDNMT2TOPOY2HRP and cloned in pENTR/D–TOPO vector (Invitrogen; **Table S1**). The fragment was then shuttled into the pGADT7 destination vector [pGADT7-GW (Clontech laboratories Inc. USA)] using the Gateway LR Clonase™ II enzyme mix (Invitrogen, USA) following manufacturer’s instructions. Bait construct was prepared using the yeast expression vector pGBKT7 (Clontech laboratories Inc. USA). *Pp3c9_25690V3.1* coding fragment (621 bp) was amplified using cDNA prepared from total RNA isolated from protonema tissue subjected to 400 mM NaCl stress for 24 h. Gene-specific primer pair Pp3c9_25690Y2H_FP and Pp3c9_25690Y2H_RP were used for PCR. The amplicon was digested with and ligated to *Sal*I and *Pst*I digested pGBKT7 vector. The construct preparations were validated by restriction enzyme digestion and DNA sequencing.

### Yeast Two-Hybrid Assay

Yeast transformations were performed using the EZ-Yeast transformation kit (MP Biomedicals, USA) as per manufacturer’s recommendations. Briefly, 2 µg each of pGADT7 (AD) and pGBKT7 (BD) fusion constructs were co-transformed in yeast AH109 suspended in 125 µl EZ-Transformation solution along with 5 µl carrier DNA. The mixture was incubated at 42°C for 30 min. The cells were then plated on Synthetic Dropout/-Leucine/-Tryptophan, SD/-Leu/-Trp (SD-LW) plates and incubated at 30°C for 3–4 days. Transformants showing robust growth were then streaked on SD/-Leu/-Trp/-Histidine (SD-LWH), SD/-Leu/-Trp/-His/-Adenine (SD-LWHA) and SD-LWHA + X-*α*-Gal synthetic dropout solid media plates for selection of transformants displaying protein–protein interactions.

## Results

### Identification of Differentially Expressed Genes in *PpDNMT2* Mutant

**Figure 1:**
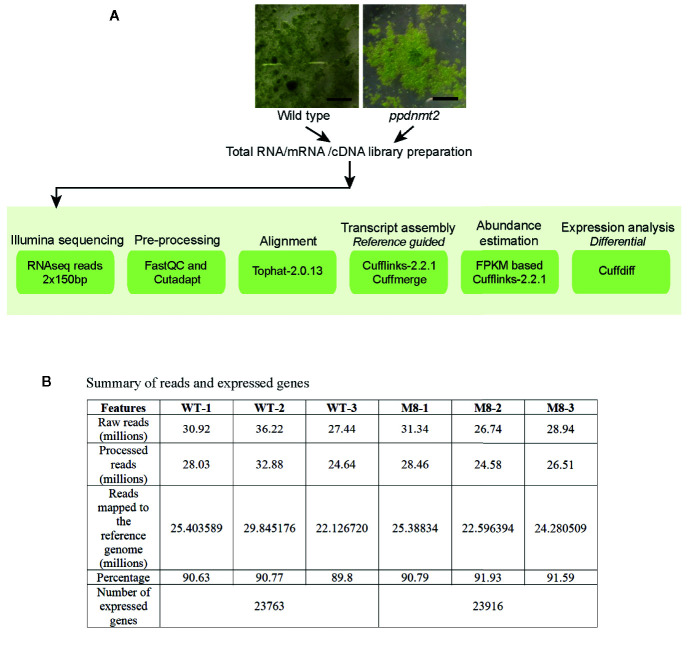
**(A)** Outline of RNA sequencing strategy employed for identification of differentially expressed genes in *PpDNMT2* knockout plants**. (B)** Table summarizing statistics of reads generated in sequencing and their assembly. Scale bar = 1 cm.

**Figure 2:**
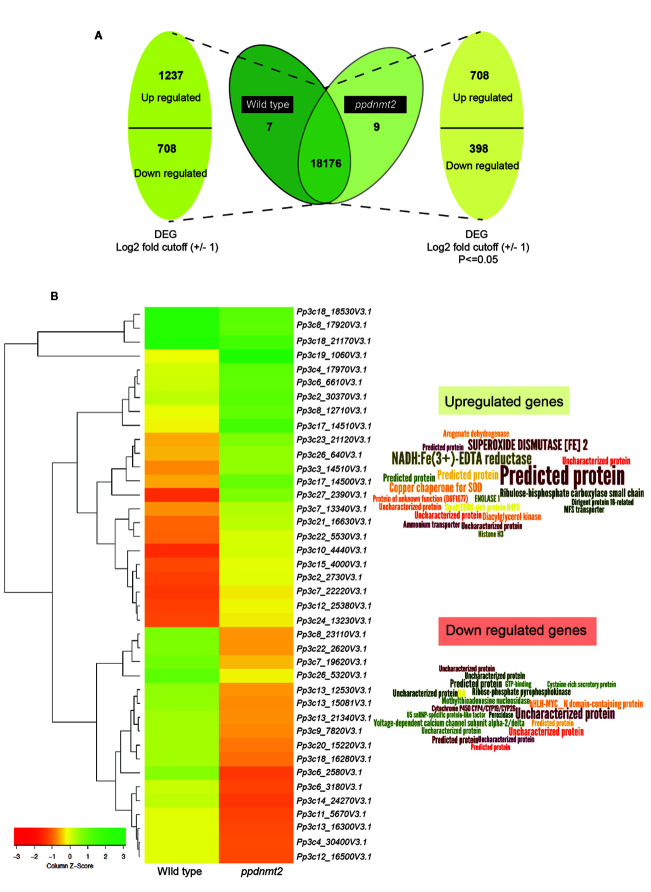
**(A)** Representation of differentially expressed genes with numbers denoting upregulated/downregulated genes and those expressing in common and exclusively in wild type and *ppdnmt2#8*, respectively. **(B)** Left: Hierarchical clustering analysis of top 20 up and downregulated genes displayed in the form of a heat map where green color shows upregulated and red depicts downregulated genes. Right: Word cloud representation of top 20 differentially expressed genes in *ppdnmt2* where word size corresponds to Log_2_ changes in transcript abundance (log_2_ foldchange_mutant/wild type_). Word clouds have been generated using Wordle.net.in using advanced settings.

To gain insight into the molecular effects of loss of *PpDNMT2* function, differentially expressed genes in *ppdnmt2#8* were identified by comparing its transcriptome with wild type plants using reference-based transcriptome method. We had previously characterized *ppdnmt2#8* (here after referred to as *ppdnmt2)* and shown that these plants were highly sensitive to salt and mannitol stress (Arya et al., 2016). Using the experimental pipeline for RNAseq outlined in **Figure 1A**, a total of 181.60 million Illumina HiSeq reads were generated for both sample types by RNA sequencing out of which 165.09 million high quality adapter free processed reads were subjected to downstream analysis. On an average 90.91% of the reads in each biological replicate aligned to the reference genome (**Figure 1B**). Next, to correct biasness in gene length and sequencing depth, the data was normalized using FPKM metrics. Normalized read values obtained for all the biological replicates of control and *ppdnmt2* are summarized in **Table S2**. Applying a log2 fold cut-off (+/−1), 1,237 upregulated genes and 708 downregulated genes were identified in *ppdnmt2*, with seven genes expressing only in wild type and nine expressing only in *ppdnmt2* (**Figure 2A**). Out of these up and downregulated genes, 708 out of 1237 and 398 out of 708 genes were found to be P-significant with p-value <= 0.05 (**Table S3**).

Heat map of top 20 up and downregulated genes shows differential accumulation of transcripts in wild type and *ppdnmt2* (**Figure 2B**). Among the genes upregulated in *ppdnmt2* are those encoding the membrane localized oxidoreductase NADH : Fe(3^+^)-EDTA reductase (*Pp3c27_2390V3.1*) that catalyzes reduction of ferric ions (Fe3^+^) into the useful ferrous form; chloroplast localized SUPEROXIDE DISMUTASE [Fe], Fe-SOD (*Pp3c17_14510V3.1*) the antioxidant enzyme catalyzing dismutation of superoxide radicals to water and molecular oxygen (Mittler et al., 2004); copper chaperone for SOD (*Pp3c17_14500V3.1*) known to be involved in trafficking copper ions from cytosol to chloroplasts for photosynthesis (Yamasaki et al., 2008); Ribulose-bisphosphate carboxylase (*Pp3c3_14510V3.1*) that catalyzes formation of 3-phosphoglycerate from carbon dioxide and ribulose bisphosphate in the Calvin-Benson cycle; Arogenate dehydrogenase (*Pp3c12_25380V3.1)* that catalyzes oxidative decarboxylation of arogenate leading to biosynthesis of the two aromatic amino acids Tyrosine and Phenylalanine (Rippert and Matringe, 2002a; Rippert and Matringe, 2002b); the glycolytic enzyme Enolase (*Pp3c15_4000V3.1*) that catalyzes synthesis of phosphoenolpyruvate that serves as precursor for biosynthesis of aromatic amino acids in chloroplasts; membrane proteins involved in nitrogen uptake in the form of nitrate/nitrite (nitrate/nitrite porter, NNP family of Major Facilitator Superfamily, MFS; *Pp3c7_13340V3.1*) and the ammonium transporter proteins of AMT family (*Pp3c22_5530V3.1*); Dirigent protein-like (*Pp3c6_6610V3.1*) known to be involved in defense response by activating formation of lignin-like polymers in *P. patens* (Reboledo et al., 2015; **Figure 2B** right panel, **Table S3**).

On the other hand, the top 20 downregulated genes include those encoding the bHLH-MYC transcription factors (*Pp3c6_3180V3.1*) that are possibly involved in plant growth, development, and stress response, R2R3-MYB transcriptional repressor (*Pp3c6_3186V3.1*) regulating phenylpropanoid pathways and lignin biosynthesis (Ma and Constabel, 2019); ribose-phosphate pyrophosphokinase (*Pp3c8_17920V3.1*), a known component of the multisubunit E3 ubiquitin ligase that forms anaphase promoting complex/cyclosome involved in phytohormone regulation (Yu et al., 2017); voltage-gated calcium channel proteins (*Pp3c7_19620V3.1*); Methylthioadenosine nucleosidase (*Pp3c26_5320V3.1*) that plays key roles in methionine recycling pathway essential in protein and S-adenosyl methionine synthesis and that serves as substrate for ethylene biosynthesis (Bürstenbinder et al., 2010); the Cytochrome P450 (*Pp3c9_7820V3.1*) possibly involved in phytohormone biosynthesis; the antioxidant enzyme peroxidase (*Pp3c12_16500V3.1*) and U5 SnRNP-specific protein (*Pp3c13_21340V3.1*) that functions as E3 ubiquitin-protein ligase RFWD2 that plays an important role in protein degradation *via* the ubiquitin proteasome system (**Figure 2B** right panel, **Table S3**).

**Figure 3:**
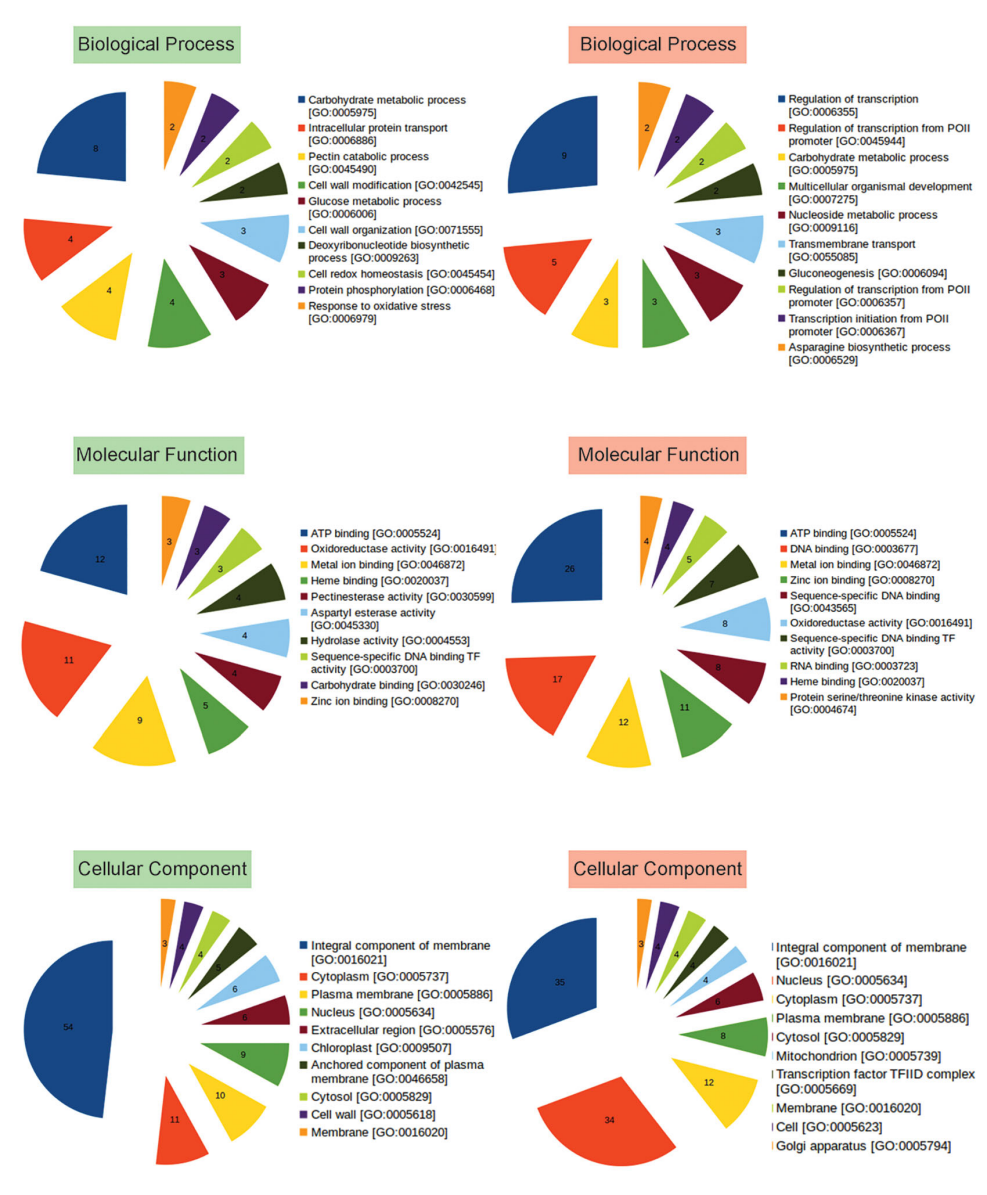
Representation of functional classification of up and downregulated genes into Biological Process, Molecular Function, and Cellular Component categories by GO analysis. Number in each pie represents how many times the function (BP, CC, MF) is expressed (abundance) in the data. GO based categorization of upregulated genes are labeled in green, while those of downregulated genes are labeled in red.

To identify functions of other up and downregulated genes, Gene Ontology (GO) analysis was performed, and genes belonging to different categories of Biological Processes, Molecular Functions, and Cellular Compartments were identified (**Figure 3**; **Table S4**). Majority of the upregulated genes were observed to encode membrane proteins with ATP binding, oxidoreductase, metal ion binding activities involved in carbohydrate metabolism, intracellular protein transport, cell wall modification, DNA biosynthetic process, cell redox homeostasis, protein phosphorylation, response to oxidative stress, defense response, response to light stimulus, glycogen biosynthetic process. While, the downregulated genes were those involved in transcription regulation, carbohydrate metabolism, transmembrane transport, Gluconeogenesis, mRNA processing, signal transduction, methylation, cell redox homeostasis (**Figure 3**, **Table S4**).

**Figure 4:**
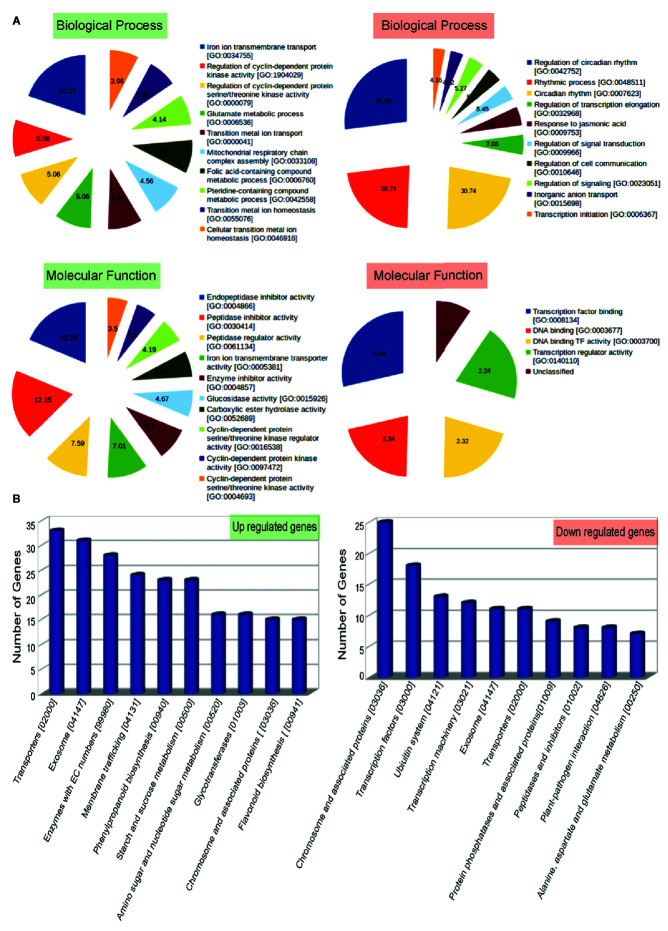
**(A)** Top 10 Gene Ontology terms of Biological Process (BP) and Molecular Functions (MF) enriched in up (labeled in green) and downregulated (labeled in red) genes. Enriched BP and MF with p value < 0.05 and with an enrichment factor > 1 are shown. Numbers represent fold change enrichment value of a particular function in each dataset. **(B)** Graphical representation of up and downregulated pathways (X-axis) with number of genes identified in each pathway category plotted on the Y-axis.

To understand the biological significance of genes mis-expressed in *ppdnmt2*, GO enrichment analysis was performed. Loss of *PpDNMT2* was observed to significantly affect molecular and physiological processes with GO related to iron ion transmembrane transport, regulation of protein kinase activities, glutamate metabolic process, mitochondrial respiratory chain complex assembly besides others, with endopeptidase inhibitor/regulator activities, Iron ion transmembrane transporter activity, etc to be enriched in the upregulated GO data set (**Figure 4**; **Table S5**), while biological processes that recur with regularity/rhythmicity or processes related to generation and maintenance of such activities, response to jasmonic acid, regulation of signal transduction and cell communication with DNA transcription activity related terms were observed to be enriched among the downregulated GO data set (**Figure 4**; **Table S6**).

Analysis of biological pathways affected by loss of *PpDNMT2* function shows pathways involving transporters, exosome pathways related to RNA degradation/mRNA surveillance, membrane trafficking, phenylpropanoid biosynthesis, starch and sucrose metabolism, pathways involving glycotransferases and chromosome associated proteins to be upregulated. While chromosomes and associated protein pathways; transcription factors, Ubiquitin system, transcription machinery, transporters, protein phosphatases, peptidases, plant pathogen interaction were downregulated (**Figure 4B**; **Table S7**).

Taken together, analysis of differentially expressed genes and associated pathways shed light on the biological processes affected by loss of *PpDNMT2* function that may correlate with the observed stress sensitivity in *ppdnmt2* mutants (Arya et al., 2016). The above results suggest that *PpDNMT2* may play a crucial role in regulating protein activities spanning diverse biological processes that directly or indirectly contribute towards maintenance of cellular homeostasis at molecular and physiological levels.

### *PpDNMT2* Regulates Stress Signal Transduction Pathways

Loss of *PpDNMT2* makes the plants sensitive to elevated salt in the growth medium (Arya et al., 2016). To understand the underlying molecular mechanism of *PpDNMT2* function in this process, we analyzed expression of genes encoding regulatory components of pathways known to be affected by salinity in *ppdnmt2*.

**Figure 5:**
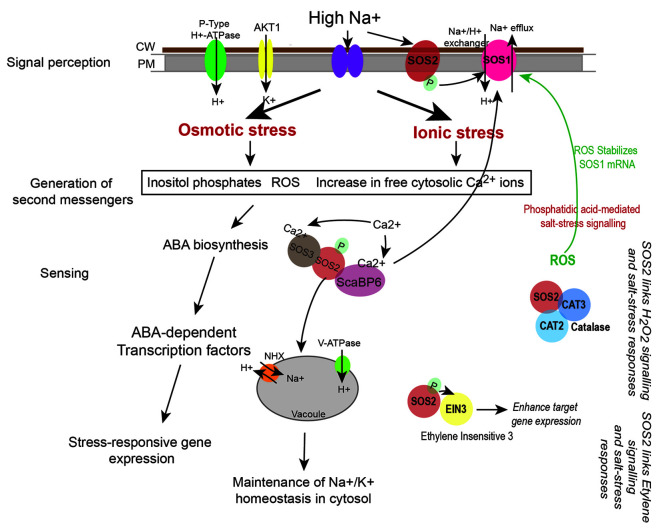
Regulation of sodium (Na^+^) and potassium (K^+^) ion homeostasis by SOS pathway. High Na^+^ ions induce osmotic and ionic stress in cells. This leads to increase in levels of cytosolic Ca^2+^, generation of reactive oxygen species (ROS), and increase in Inositol phosphates. Perturbations in Ca^2+^ levels is sensed by calcium binding proteins such as SOS3 and ScaBP6 that activate the protein kinase SOS2 thus initiating a phosphorylation cascade. This leads to activation of the plasma membrane localized Na^+^/H^+^ exchanger SOS1 (that effluxes Na^+^ from the cytoplasm) and the NHX exchanger on the vacuoles (that accumulate Na^+^ in the vacuole) thus leading to maintenance of ion homeostasis in cells. Salt stress response is also linked to hydrogen peroxide (H_2_O_2_) signaling as SOS2 directly interacts with CATALASE (CAT2 and CAT3), while its interaction with Ethylene Insensitive 3 (EIN3) links SOS pathway to ethylene signaling processes (Verslues et al., 2007; Quan et al., 2017). ROS and stress responsive changes in gene expression may also lead to biosynthesis of phytohormones such as ABA that can amplify the stress signal by activating ABA-dependent transcription factors that lead to expression of stress-responsive genes.

Salt stress disrupts ionic equilibrium in cells due to accumulation of excess toxic Na^+^ in the cytoplasm and deficiency of essential ions such as K^+^. This results in activation of the conserved Salt Overly Sensitive (SOS) pathway that functions to regulate sodium ion homeostasis (Zhu, 2002). Salt stress also activates osmotic stress signaling pathways and Abscisic acid (ABA)-dependent pathway that play key roles in osmotic adjustment and activation of transcription factors that regulate expression of stress-responsive genes necessary for stress tolerance/adaptation (**Figure 5**). Cell wall and plasma membrane play an important role in perception and transmission of stress signals and in plant defence response. Sodium ions induce biochemical changes in cell membranes that lead to alterations in their physical properties (López-Pérez et al., 2009; Moura et al., 2010; Ji et al., 2013; Van der Does et al., 2017). This triggers generation of secondary messengers such as cytosolic Ca^2+^, Reactive Oxygen Species (ROS) and Inositol phosphates (**Figure 5**). Increased cytosolic Ca^2+^ are sensed by calcium sensors such as the EF (Extra Finger) hand-type calcium binding protein SOS3 that binds to and activates the Serine/Threonine kinase SOS2. SOS2 then phosphorylates the plasma membrane localized Na^+^/H^+^ antiporter SOS1 and the SOS3-LIKE CALCIUM BINDING PROTEIN 8 (SCaBP8) resulting in activation of the high affinity K^+^ channel proteins (such as AKT1) that begin export of Na^+^ from the cytoplasm and increase influx of K^+^ (**Figure 5**). SOS2 also activates the vacuolar Na^+^/H^+^ exchanger (NHX) for compartmentalization of Na^+^ into the vacuole (Qiu et al., 2004; Batelli et al., 2007). Increased levels of ROS during this process can damage proteins, lipids, DNA, and carbohydrates (Miller et al., 2010). To mediate ROS removal, cells activate both enzymatic and non-enzymatic ROS scavengers. Enzymatic scavengers include SOD, Catalase (CAT), Ascorbate peroxidase (APX), monodehydroascorbate reductase (MDHAR) among others, while the non-enzymatic scavengers include ascorbic acid, alkaloids, carotenoids, and other phenolic compounds (Zhu, 2002; Yang and Guo, 2018).

**Figure 6:**
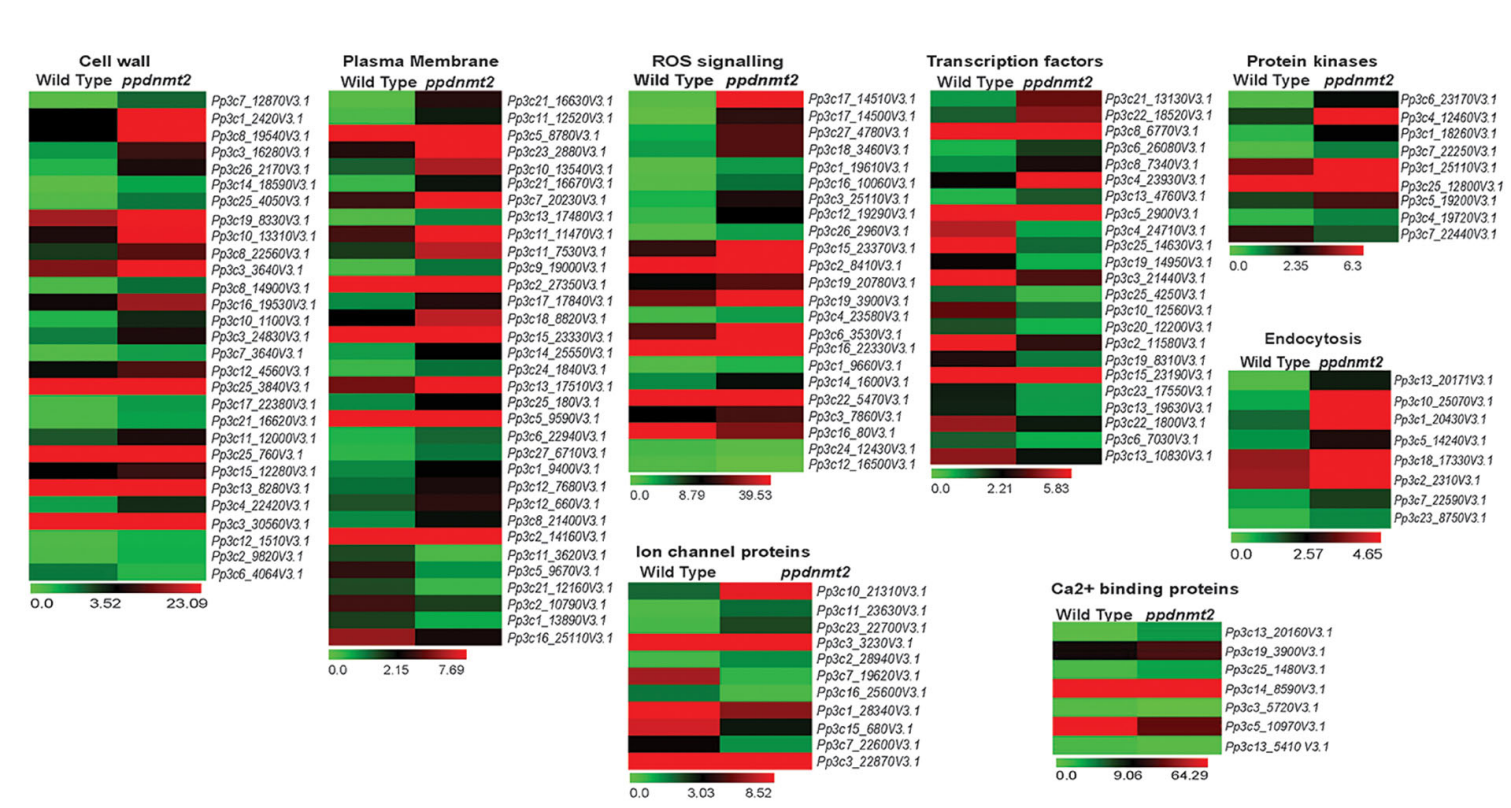
Expression patterns of genes encoding proteins involved in stress sensing at cell wall and plasma membrane, ion homeostasis regulation, ROS signaling, cytoskeletal dynamics, and those encoding transcription factors. Average FPKM values of genes in wild type and *ppdnmt2* are presented by cluster display (heat map) with color scale representing range of expression values shown at the bottom of each heat map.

Transcriptome landscape of *ppdnmt2* reveals many genes involved at different levels of stress signal transduction pathways to be differentially expressed. At the level of cell wall, genes involved in cell wall biosynthesis and maintenance such as those encoding cellulose synthase, expansins, cross-linking glycans (Xyloglucan : Xylosyl transferases), glycoproteins (Arabinogalactans), and pectinesterases were observed to be upregulated (**Figure 6**; **Table S3**). While at the plasma membrane, genes involved in lipid metabolic process, lipid transport, fatty acid biosynthetic process, diacylglycerol were upregulated while those encoding lipid binding proteins were downregulated in *ppdnmt2* (**Figure 6**; **Table S3**). Among the genes encoding Ca^2+^ ion binding proteins, calmodulin encoding genes (*Pp3c25_1480V3.1* and *Pp3c14_8590V3.1*) were observed to be upregulated while SOS3 (*Pp3c13_5410V3.1*), the key regulator of the SOS pathway was downregulated in *ppdnmt2*. The Leucine-rich repeat receptor-like protein kinases (LRR-RLK) are membrane localized receptor proteins that sense and transduce signals downstream by autophosphorylation followed by phosphorylation of specific substrates. They are known to play crucial roles in stress response and plant development (Liu et al., 2017). Out of 119 genes encoding LRR-RLK in *P. patens*, eight were upregulated in *ppdnmt2* (*Pp3c6_23170V3.1*, *Pp3c1_18260V3.1*, *Pp3c7_22250V3.1*, *Pp3c1_25110V3.1*, *Pp3c25_12800V3.1*, *Pp3c5_19200V3.1*, *Pp3c4_19720V3.1*), while CPK13 homolog of calcium-dependent protein kinase (*Pp3c7_22440V3.1*) known to inhibit K(^+^) channel proteins KAT2 and KAT1 in Arabidopsis was downregulated (Ronzier et al., 2014; Liu et al., 2017). Expression of genes encoding Fe-SOD (*PpFeSD3*; *Pp3c17_14510V3.1*) was significantly upregulated along with those encoding peroxidases (*Pp3c1_19610V3.1*, *Pp3c16_10060V3.1*; *Pp3c12_19290V3.1*, *Pp3c26_2960V3.1*, *Pp3c19_20780V3.1*) and the non-enzymatic ROS scavenger ascorbic acid, monodehydroascorbate reductase (MDHAR, *Pp3c2_8410V3.1*) and Glutathione S-transferase (GST, *Pp3c22_5470V3.1*), while those encoding lactoylglutathione lyase glyoxalase I (*Pp3c24_12430V3.1*) were downregulated in *ppdnmt2* (**Figure 6**; **Table S3**).

Genes involved in maintaining ion homeostasis were also observed to be differentially expressed in *ppdnmt2*. Genes encoding copper ion transmembrane transporter (solute carrier family 31; *Pp3c27_4780V3.1*) and the copper chaperone for SOD (*Pp3c17_14500V3.1*) and ATOX1 chaperone (*Pp3c14_1600V3.1*) were observed to be significantly upregulated in *ppdnmt2*. Expression of three out of 22 Plastocyanin genes encoded in *P. patens* genome (*Pp3c3_25110V3.1*, *Pp3c6_3530V3.1*, *Pp3c16_22330V3.1*) that require copper for their activity were also observed to be upregulated in *ppdnmt2*. This suggests that *PpDNMT2* may play a crucial role in Cu^2+^ trafficking pathway from its point of entry in the plasma membrane to its delivery in the chloroplasts. Further, four genes encoding Aquaporins (*Pp3c10_21310V3.1*, *Pp3c11_23630V3.1*, *Pp3c3_3230V3.1*), the membrane bound channel proteins that facilitate transport of water and small solutes across plasma membrane; genes encoding potassium channel (AKT1-related, *Pp3c2_28940V3.1*) proteins were also observed to be upregulated in *ppdnmt2*.

Salt stress is also known to increase endocytosis and active vesicle movement that leads to Na^+^ accumulation in vacuoles (Hamaji et al., 2009). Genes encoding proteins regulating membrane vesicle trafficking such as Clathrin (*Pp3c13_20171V3.1*), ADP-ribosylation factor 1 (ARF1, *Pp3c10_25070V3.1*, *Pp3c1_20430V3.1*), Secretory Carrier-Associated Membrane Proteins (SCAMPs, *Pp3c5_14240V3.1*), Soluble N-ethylmaleimide-sensitive factor attachment protein receptors (SNARE, *Pp3c18_17330V3.1*) were observed to be upregulated in *ppdnmt2* (**Figure 6**; **Table S3**).

Cytoskeleton dynamics regulated by microtubules, microfilaments, and their associated proteins also play crucial roles in stress tolerance in plants. Depolarization of microtubules followed by their repolarization is critical for stress tolerance (Wang et al., 2007). Loss of *PpDNMT2* function results in upregulation of Microtubule-localized SPIRAL1 protein encoding gene (*Pp3c22_7610V3.1*; **Figure 6**, **Table S3**). These proteins are known to be required for anisotropic root growth in rapidly elongating cells in Arabidopsis (Shoji et al., 2004; Nakajima et al., 2006).

A number of genes encoding transcription factors that affect stress responsive gene expression and other related functions were also observed to be differentially expressed in *ppdnmt2*. Genes encoding the APETALA2/ETHYLENE-RESPONSIVE FACTOR (AP2/ERF) were both up and downregulated (upregulated: *Pp3c21_13130V3.1*, *Pp3c6_26080V3.1*, *Pp3c8_7340V3.1*, *Pp3c4_23930V3.1*, *Pp3c5_2900V3.1*; down: *Pp3c22_1800V3.1*, *Pp3c2_11580V3.1*, *Pp3c25_4250V3.1*, *Pp3c19_14950V3.1*, *Pp3c4_24710V3.1*). AP2/ERF forms a large group of plant-specific transcription factors that activate genes in response to ABA, cold, drought, and salinity. They also regulate chloroplast division under salt stress in *P. patens* (Mizoi et al., 2012; Do et al., 2020). Plant-specific Plant AT-rich sequence-and zinc-binding transcription factor (PLATZ) transcription factors were also differentially expressed (upregulated: *Pp3c13_4760V3.1*; Downregulated: *Pp3c15_23190V3.1*, *Pp3c10_12560V3.1*). These proteins function in RNA polymerase III transcription machinery to regulate biogenesis of tRNA and 5S rRNAs in maize (*FL3*) and rice (*GL6*) that affects endosperm storage filling (Li et al., 2017) and rice grain length and number (Wang and Schippers, 2019). Two out of 22 small Heat Shock proteins (sHSPs) encoded in *P. patens* genome were also differentially expressed (upregulated: *Pp3c8_6770V3.1* and *Pp3c25_14630V3.1*: downregulated). sHSPs are known to prevent protein aggregation and facilitate protein refolding by chaperons during stress and are required for stress recovery in *P. patens* (Ruibal et al., 2012). *Pp3c22_18520V3.1* encoding the sigma factor (SigE) of the plastid RNA polymerase, a multisubunit bacteria-type enzyme in chloroplasts was observed to be upregulated in *ppdnmt2*. The plastid sigma factors are known to integrate clock and light regulatory mechanisms that underly daily expression patterns of plastid genes in *P. patens* (Ichikawa et al., 2004). Three genes (*Pp3c13_10830V3.1*, *Pp3c20_12200V3.1* and *Pp3c3_21440*) encoding WRKY transcription factors were observed to be downregulated in *ppdnmt2*. These transcription factors form important components of stress signaling cascades and signaling in response to internal developmental cues in plants (Bakshi and Oelmüller, 2014). *PpDNMT2* may also affect plant-specific phytohormone signaling responses. Genes involved in cytokinin metabolism (degradation, activation, signaling; *Pp3c6_7030*, *Pp3c23_17550*, and *Pp3c13_19630*); DELLA protein encoding genes (*Pp3c19_*8310) known to function as negative regulators of gibberellin signaling and in integration of multiple hormone signaling pathways were observed to be downregulated (Davière and Achard, 2016) (**Figure 6**; **Table S3**). Differential expression of genes involved in phytohormone signal transduction may explain the abnormal branching pattern observed in *ppdnmt2* (Arya et al., 2016).

The above results therefore suggest that *PpDNMT2* may play a pivotal role in maintaining ion homeostasis by regulating genes involved in stress signaling pathways and in plant development by affecting genes involved in phytohormone signaling in *P. patens*.

### Identification of Proteins Co-Immunoprecipitating With PpDNMT2

**Figure 7:**
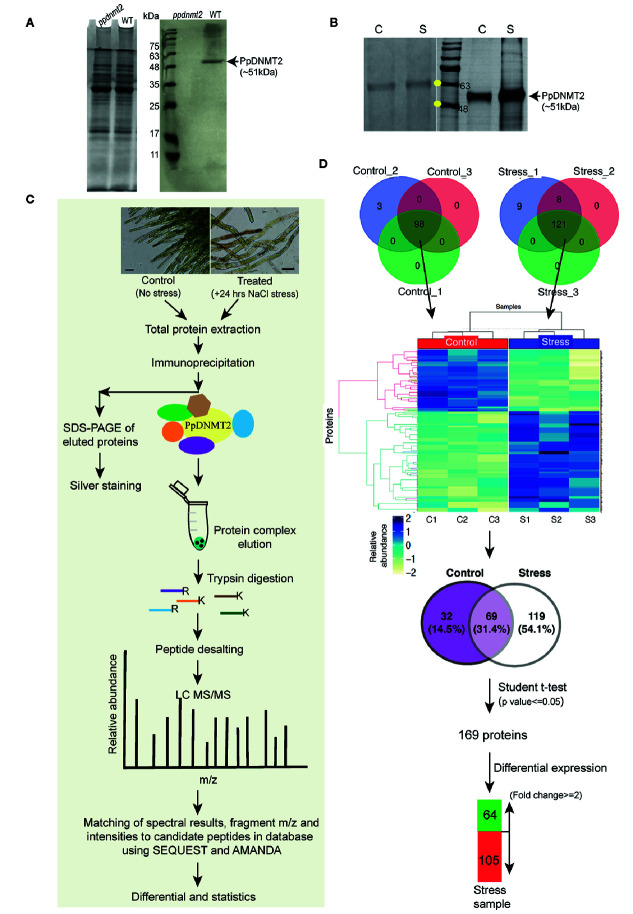
**(A)** (Left) Coomassie stained image of polyacrylamide gel showing fractionation of equal amounts of total protein (50 µg each) extracted from *ppdnmt2* and wild type protonema tissue. (Right) Western blot probed with PpDNMT2-specific antibody showing presence of PpDNMT2 protein band of expected size in wild type plants and its absence in *ppdnmt2*. **(B)** (Left) Silver stained image of polyacrylamide gel showing proteins isolated from control (C, 0 h) and stress-treated, (S) protonema tissue. (Right) Immunoblot of proteins probed with PpDNMT2-specific antibody. Arrow points to the band position of ~51 kDa PpDNMT2, and the yellow dots on the marker bands in the protein ladder indicate 48 and 63 kDa proteins, respectively. **(C)** Outline of steps followed to immunoprecipitate proteins in complex with PpDNMT2 followed by their identification by LC-MS/MS. Scale bar = 50 µm. **(D)** Summary of analyzed data. Venn diagram on top shows number of proteins identified in each of the three biological replicates of control and stress samples. Distribution of these proteins in both samples before and after application of Student t-test. Heat map showing differential abundance of 169 proteins in the Control (C1–C3) and Stress (S1–S3) samples. For generating heat map abundance, values were log2 transformed and z-normalized. Hierarchical clustering was then performed using Euclidian distance and average linkage using in-House R script (Package: ComplexHeatmap). Pale yellow and blue colors, respectively, represent low and high protein abundance.

To gain insight into the interactome of PpDNMT2, immunoprecipitation coupled with Mass spectrometry (IP-MS) was performed. First, to check specificity of the PpDNMT2 antibody, a western blotting was performed using equal amounts of total protein extracted from wild type and *ppdnmt2* protonemata tissue and probed with anti-PpDNMT2-HRP conjugate. A single protein band of expected size (~51 kDa) corresponding to PpDNMT2 was detected in wild type samples and not in *ppdnmt2* indicating the antibody prepared was specific for PpDNMT2 (**Figure 7A**). Next, to select the conditions under which *PpDNMT2* is optimally expressed, we took note of the fact that among all the cytosine methyltransferases expressing in protonema, *PpDNMT2* expression has been observed to be the weakest (Malik et al., 2012). We have also previously shown that *PpDNMT2* is differentially expressed under salt and mannitol stress with transcript levels decreasing under stress conditions (Arya et al., 2016). To analyze the expression at protein level under stress, we performed western analysis using total protein extracted from control protonema (non-stress) and protonema exposed to 24 h salt stress (referred to as treated from here on) and probing with anti-PpDNMT2-HRP. Low levels of PpDNMT2 were detected in both control and treated samples. To assess the levels of endogenous PpDNMT2 more clearly we enriched the target protein by affinity purification in each sample type and then performed western blotting. Strong induction in PpDNMT2 expression was observed in samples harvested after 24 h stress treatment in comparison to control (**Figure 7B**). Hence, we selected 0 and 24 h stress as the time points for protein extraction and affinity purification. The interacting protein partners in the immune complex were then identified by LC-MS. IP procedure with beads devoid of antibody was not included in this study.

Following the experimental pipeline outlined in **Figure 7C**, a total of 101 proteins were identified in control out of which 98 were present in all three biological replicates. Similarly, a total 188 proteins were identified in treated samples out of which 121 were common in all the three replicates (**Figure 7D**). Strong correlation among the biological replicates of control and treated samples with Pearson correlation coefficient >= 0.8 among C1–C3 (control) and >=0.7 between S1–S3 (treated) was observed (**Figure S1**). Heat map generated using abundance values of P-significant proteins show differential accumulation of proteins under control and stress conditions. Out of 98 and 121 proteins identified in the control and treated samples respectively, 69 were common to both control and treated while 32 were present only in the control and 119 only in the stress samples. After statistical analysis applying p value <= 0.05 and fold change >= 2 cut-off, 169 proteins were shortlisted of which 64 proteins were observed to be present in >=2 fold abundance while 105 proteins below this cut-off in treated sample (**Figure 7D**, **Table S8**).

**Figure 8:**
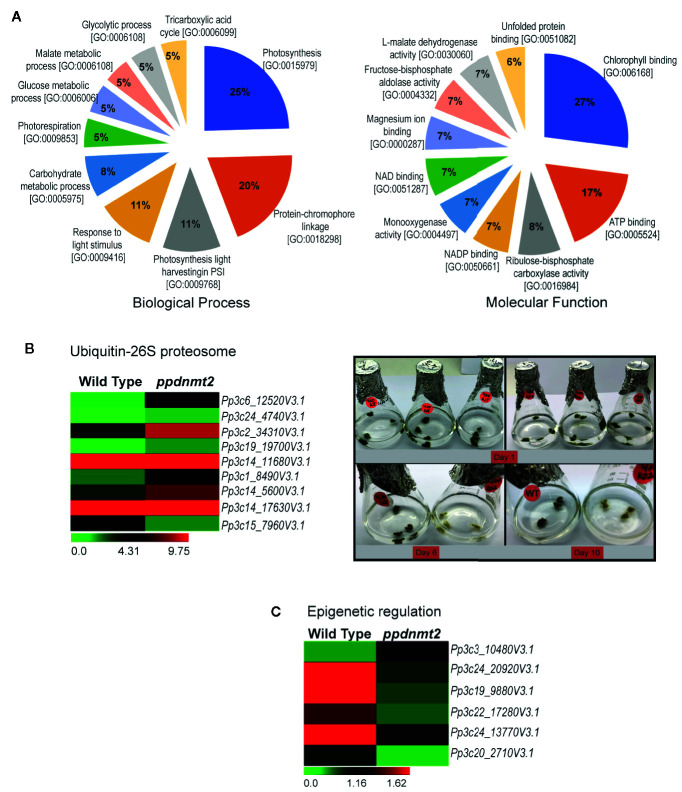
**(A)** Pie charts depicting top 10 Biological Process and Molecular Function Gene Ontology categories to which P-significant (p value <= 0.05) proteins co-immunoprecipitated with PpDNMT2 can be classified. **(B)** Left: Differential expression pattern of genes involved in Ubiquitin-26S proteasome in wild type and *ppdnmt2* presented by cluster display with color scale representing FPKM expression values; Right: Wild type (WT) and *ppdnmt2* (KO#2) protonemata propagated in BCDAT media supplemented with 400 mM NaCl under similar growth conditions for 10 days to analyze stress tolerance of plants lacking *PpDNMT2* function. **(C)** Differential expression of genes in wild type and *ppdnmt2* involved in epigenetic regulation displayed in the form of a heat map with color scale representing range of FPKM values of the selected genes.

To gain insight into biological processes, molecular function and cellular localization of proteins co-immunoprecipitated with PpDNMT2, GO analysis was performed. GO terms with photosynthesis, protein-chromophore linkage (Biological process) and chlorophyll binding, ATP binding (Molecular function) affiliation were observed to be enriched. Though annotations for a number of proteins were not available in public databases, on the basis of available information, it was observed that majority of the proteins were integral components of membranes/chloroplast thylakoid membranes involved in photosynthesis, response to light stimulus, carbohydrate metabolism, photorespiration, glycolytic process with chlorophyll, or ATP binding activities (**Figure 8A**). High abundance of proteins related to chloroplast and photosynthesis is most likely a reflection of abundant chloroplasts in chloronema tissue used for proteome analysis.

### PpDNMT2 Regulates Ubiquitin-26S Proteasome Degradation Pathway and Epigenetic Regulatory Pathway Genes

Comparison between the gene set affected by loss of *PpDNMT2* and the co-IP protein data reveals novel roles for *PpDNMT2* in *P. patens*. Among the proteins identified in PpDNMT2 immunocomplex under both control and salt stress was RPT4, one of the six AAA-ATPases of the 19S regulatory particle of the Ubiquitin-26S proteasome system that regulates recognition and unfolding of substrates during protein degradation (**Table S8**). Our transcriptome dataset also revealed genes of the ubiquitin-26S degradation pathway to be differentially expressed. Genes encoding the ubiquitin-conjugating enzyme E2C (*Pp3c6_12520V3.1*), E3 ubiquitin ligase MIEL1 (*Pp3c14_5600V3.1*), proteins with ubiquitin transferase (*Pp3c24_4740V3.1*, *Pp3c19_19700V3.1*, *Pp3c1_8490V3.1*) and ligase (*Pp3c2_34310V3.1*) activities, a subunit of the 19S regulatory particle involved in substrate recognition and unfolding (Rpn12) and the 26S proteasome regulatory subunit N12 (*Pp3c14_17630V3.1*) were upregulated, while the E3 ubiquitin-protein ligase MARCH6 (*Pp3c15_7960V3.1*) was downregulated in *ppdnmt2* (Wang and Schippers, 2019; **Figure 8B** left panel; **Table S3**). To experimentally check if loss of *PpDNMT2* function does affect protein degradation/senescence in response to salt stress, wild type and *ppnmt2* were propagated in BCDAT media supplemented with high NaCl under similar growth conditions. Early senescence in *ppdnmt2* (within 6 days) observed by visual discoloration of *ppdnmt2* protonema in comparison to wild type plants was observed indicating low stress tolerance of *ppdnmt2* plants (**Figure 8B**, right panel). This suggests a role for *PpDNMT2* in sensing/maintaining cellular proteostasis by affecting expression of Ubiquitin-26S proteasome pathway genes.

In the co-IP protein set, four histone 4 proteins (*Pp3c27_7460V3.1*, *Pp3c14_26430V3.1*, *Pp3c22_22190V3.1* and *Pp3c21_19690V3.1*) known to be core components of nucleosomes were identified with high confidence in treated samples (**Table S8**). Our transcriptome data set also revealed that genes encoding proteins involved in histone modification are differentially expressed. Three genes encoding histone methyltransferases were downregulated in *ppdnmt2*. These include *Pp3c24_20920V3.1* encoding the SET domain containing histone lysine N methyltransferase and *Pp3c19_9880V3.1 and Pp3c22_17280V3.1* encoding the homolog of Arabidopsis *SUVR5* that mediates H3K9me2 methylation and is required for transcriptional silencing (Caro et al., 2012). The homolog of Arabidopsis transcriptional repressor *SIN3* (a component of Histone deacetylase complex) encoded by *Pp3c24_13770V3.1* was also downregulated in *ppdnmt2*, while *Pp3c3_10480V3.1* encoding the RNA-dependent DNA polymerase was upregulated. It was also observed that *Pp3c20_2710V3.1* encoding gag protein was activated in *ppdnmt2* (**Figure 8C**; **Table S3**). Transcription of GAG structural proteins encoded by the long terminal repeat (LTR) copia-type retrotransposon is the first/obligatory step required for mobility and transposition of repetitive elements. Hence, transcription of *gag* in *ppdnmt2* and not in wild type suggests active transposition of this class of retrotransposons in cells and a role for *PpDNMT2* in silencing LTR copia type retrotransposons. Our previous observation that PpDNMT2 localizes both in the nucleus and the cytoplasm further supports its role in epigenetic silencing of TE observed in this study (Malik et al., 2012). Taken together, our proteomic and transcriptome data suggests a key role for *PpDNMT2* in chromatin and epigenetic gene regulation in *P. patens*.

### PpDNMT2 Interacts With Superoxide Dismutase

To shortlist putative interactors of PpDNMT2 for protein interaction validation we noted a close link between PpDNMT2 and SOD from our transcriptome and interactome studies. Our transcriptome data shows strong upregulation of *PpFSD3* (*Pp3c17_14510V3.1*) encoding Fe-SOD in *ppdnmt2* (5.9 folds). Target P2.0 (Emanuelsson et al., 2000) and ChloroP1.1 (Emanuelsson et al., 1999) servers predict PpFSD3 to be localized to the chloroplast. Interestingly, among the proteins existing in PpDNMT2 immunocomplex, four CuZn-SODs were identified in the treated samples (**Table S8**). Two of these CuZn-SODs, PpCSD1 (accession#A9SX31; *Pp3c9_24840*) and PpCSD2 (accession# A9SX65; *Pp3c9_25690*) have been previously described (Higashi et al., 2013). Expression of genes encoding PpCSD1 and PpCSD2 is known to be repressed under copper deficient conditions. Downregulation of CuZn-SODs is known to be functionally compensated by increase in Fe-SOD levels as an adaptive strategy in higher plant chloroplasts to mitigate harmful effects of ROS under copper limiting conditions in chloroplasts (Yamasaki et al., 2008). These observations suggested a close link between PpDNMT2 and the antioxidant enzyme activities. To strengthen this hypothesis we performed directed yeast two hybrid assay to study interaction between PpDNMT2 and PpCSD2 (*Pp3c9_25690V3.1*) in yeast nucleus.

**Figure 9:**
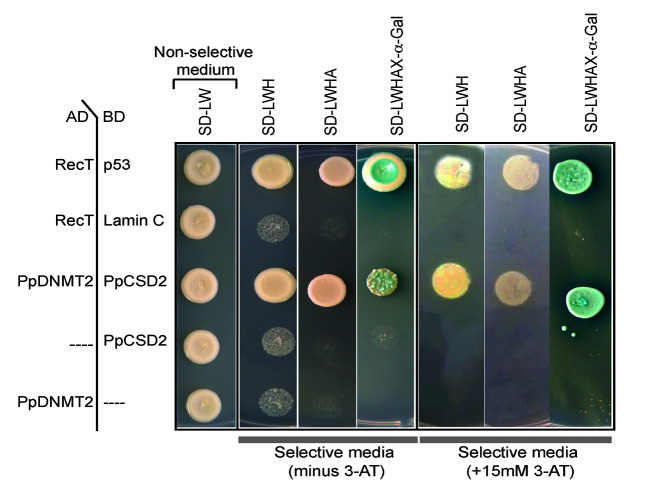
Yeast two-hybrid assay showing direct interaction between PpDNMT2 and PpCSD2. PpDNMT2 and the SV40 large T antigen (RecT) are expressed as GAL4-AD fusion protein while p53, human Lamin C, and the bait protein (PpCSD2) are expressed as GAL4 DNA binding domain (BD) fusion proteins. Non-selective and Selective synthetic dropout media lacking leucine (L), Tryptophan (W), Histidine (H) and Adenine (A) are mentioned above.

Complementary DNA (cDNA) encoding PpCSD2 was fused with GAL4 DNA binding domain (BD) and used as bait, while PpDNMT2 fused with GAL4 Activation domain (AD) was used as prey. Co-expression of the fusion proteins in yeast and growth of colonies on selective media lacking leucine, tryptophan, histidine (SD/LWH), and Adenine (SD/LWHA) indicated interaction between the bait and prey proteins *in vivo* (**Figure 9**). Further validation was performed by streaking the positive colonies on quadruple dropout media supplemented with the chromogenic substrate X-*α*-Gal (SD/LWHA X-*α*-Gal). Development of blue color in colonies indicated activation of the endogenous reporter *MEL1* by the reconstituted GAL4 as a result of interaction between PpDNMT2 and PpCSD2. SV40 large T-antigen (RecT) co-expressed with human Lamin C did not show interaction as expected and was used as negative control, while growth of colonies harboring RecT co-expressed with p53 confirmed the known interaction between RecT and p53 and was used as positive control. AD-PpDNMT2 co-expressed with empty BD-vector and BD-prey proteins co-expressed with empty AD-vectors did not result in any yeast growth and were used as controls for monitoring specificity of interaction. Strength of interaction between PpDNMT2 and PpCSD2 was also assessed by adding optimized concentration (15 mM) of 3-AT (3-amino-1,2,4-triazole), a competitive inhibitor of the His3 protein in SD-LWH selective media. Optimum growth of yeast co-expressing PpDNMT2 and PpCSD2 fusion proteins on 3-AT supplemented selective media indicated strong interaction between the two proteins (**Figure 9**).

The above results therefore show that PpDNMT2 can exist in complex with CuZn-SOD *in vivo*, and together these proteins may play a pivotal role in navigating cellular response during stress recovery.

## Discussion

The biological significance and molecular mechanism of cytosine DNA/tRNA methyltransferase *DNMT2* have remained elusive in land plants. In this study, an attempt has been made to understand the molecular mechanism of *PpDNMT2* function that is crucial for stress recovery in *P. patens* (Arya et al., 2016). We have analyzed the transcriptome of *PpDNMT2* knockout mutant (*ppdnmt2#8*) and identified proteins present in the PpDNMT2 immunocomplex by mass spectrometry after exposing wild type plants to salt stress conditions.

Among the cell organelles affected by salinity, chloroplasts are the most sensitive. These plastids are not only sites for photosynthetic activity, but they also participate in many metabolic processes including biosynthesis of lipids and fatty acids, aromatic amino acids and reduction of nitrites and sulphates (Baginsky and Gruissem, 2004). Due to the high rate of oxidizing metabolic activities and increased rate of electron flow during photosynthesis, chloroplasts are highly prone to ROS production. Further, salt stress also induces production of ROS. Hence, chloroplasts experience significant ROS-mediated damages under stress. Accordingly, the expression of many nuclear genes targeted to chloroplasts is known to be finely tuned under salt stress in flowering plants (Zörb et al., 2009; Fan et al., 2011; Wang et al., 2014; do Amaral et al., 2016). Also, in *P. patens* photosynthesis in leafy gametophores is known to increase under salt stress to reduce negative effects of salinity (Wang et al., 2008). The co-IP data set generated from stress-treated chloroplast-rich protonema tissue show enrichment of proteins involved in thylakoid membrane organization, PSII activity, CO_2_ assimilation, ROS scavenging, maintenance of ionic and osmotic homeostasis and protein turnover in PpDNMT2 immunocomplex. This suggests that in protonema cells too photosynthetic activity and the antioxidative enzyme activity are elevated to enhance stress tolerance and protection from oxidative damage though the possibility of non-specific contamination from chloroplast proteins due to their abundance in the chloroplast-rich tissue used for protein extraction cannot be ruled out. Our transcriptome data also shows upregulation of genes encoding ROS scavenging proteins (*Pp3c9_25690v3.1*, ~5.9 folds) in *ppdnmt2* in comparison to wild type plants propagated under similar conditions of continuous light. Continuous light conditions in some plants are known to affect photosynthesis rate and photochemical efficiency of PSII leading to increase in SOD activity (Haque et al., 2015). The tissue used for transcriptome analysis and proteomic studies was propagated on solid and in liquid media, respectively but these contained common moss media supplements. Hence, upregulation of genes encoding SOD in *ppdnmt2* and identification of SODs in PpDNMT2-immunocomplex under salt stress suggest an important role for *PpDNMT2* in stress mitigation pathways involved in minimizing ROS-mediated damages in *P. patens*.

*P. patens* genome encodes eight SOD isozymes that include four CuZn-SODs (PpCSD1–4), three Fe-SODs (PpFSD1–3) and one Mn-SOD (PpMSD) that are localized in the chloroplasts (PpCSD1, PpCSD2, PpFSD3), cytosol (PpCSD3, PpCSD4), mitochondria (PpMSD), and the apoplast (PpFSD1) (Higashi et al., 2013). The expression of genes encoding these metalloenzymes is known to be co-ordinately regulated depending on the bioavailability of metal ions. The CuZn-SOD and Fe-SOD encoding genes are known to express differentially under copper deficient conditions with expression of *PpCSD1* and *PpCSD2* repressed while that of *PpFSD3* strongly induced under copper limiting conditions in *P. patens* (Higashi et al., 2013). Copper is essential for activity of chloroplast electron carrier protein Plastocyanin that catalyzes transfer of electrons from the Cytb_6_f complex to PS1 in the Z-scheme of photosynthesis (Gorman and Levine, 1965). Chloroplasts are known to sustain photosynthesis under low copper conditions by downregulating CuZn-SOD and increasing levels of copper chaperone proteins to enable transfer of limited copper to plastocyanin in chloroplasts (Abdel-Ghany et al., 2005; Nagae et al., 2008). Further, to minimize ROS-mediated damage under these conditions, a switch from CuZn-SOD to Fe-SOD occurs that leads to increase in levels of Fe-SOD activity. Our proteome and transcriptome data suggest a similar adaptive mechanism to be functional in *P. patens* as well. We observe low levels of CuZn-SOD (*Pp3c9_24840V3.1*, *Pp3c9_25690V3.1*) in PpDNMT2 immunocomplex under salt stress. Further, *ppdnmt2* transcriptome shows increased levels of *Pp3c17_14500V3.1*, *Pp3c27_4780V3.1*, and *Pp3c17_14510V3.1* encoding the copper chaperone for SOD, the copper ion transmembrane transporter, and the chloroplast localized Fe-SOD. These observations suggest that *PpDNMT2* may play a crucial role in modulating SOD activities in response to copper availability in chloroplasts. We also show PpDNMT2 to interact with PpCSD2 in yeast nucleus. GFP-PpDNMT2 is known to be distributed both in the nucleus and cytoplasm in moss protonema cells (Malik et al., 2012). While PpCSD2 is predicted to be localized to the chloroplasts, no chloroplast transit peptide sequence is present in PpDNMT2. Since PpCSD2 was identified in PpDNMT2 immunocomplex under stress conditions, it is plausible that PpDNMT2 may get localized to the chloroplasts under stress, and the two proteins may interact in the plastids. In animal systems, GFP-Dnmt2 is known to shuttle between the nucleus and the cytoplasm in response to cellular stress. Dnmt2 is normally present in the nucleus but under stress conditions it re-localizes to cytoplasmic granules and RNA processing bodies (Thiagarajan et al., 2011).

Transcriptome profile also shows *ppdnmt2* cells to accumulate increased levels of apoptosis associated protein encoding genes and transcripts encoding Ubiquitin-26S proteasome components, while genes affecting telomere integrity are downregulated along with genes involved in signal transduction, cell–cell communication and intracellular protein transport. Though the levels of changes in gene expression is low or below threshold, the transcript signatures in *ppdnmt2* reflect a molecular environment that provides a possible explanation for early senescence and increased sensitivity of *ppdnmt2* under salt and osmotic stress. The molecular mechanism of how PpDNMT2-Fe-SOD complex allows plants to tolerate stress is not known yet. It is plausible that Fe-SOD may modulate tRNA methylation activity of PpDNMT2 to stabilize tRNAs and prevent their fragmentation under stress as has been shown in Drosophila (Schaefer et al., 2010). In the lower eukaryote Entamoeba the glycolytic protein Enolase is known to bind to DNMT2 (Ehmet) and inhibit its tRNA^Asp^ methylation activity under glucose starvation (Tovy et al., 2010).

Among the RNA methyltransferases in plants *TRM4B* loss-of-function mutants in Arabidopsis are known to be sensitive to oxidative stress. They also lack m5C sites on mRNA and non-coding RNAs and have reduced tRNA^Asp(GTC)^ stability (David et al., 2017). *PpDNMT2* loss-of-function mutants are also sensitive to salinity, and *PpDNMT2* may modulate activity of antioxidant proteins under stress as inferred from this study. We had also previously reported that transcription/stability of tRNA^Asp(GTC)^ is highly reduced under stress in *ppdnmt2*. This suggests that the stability of tRNA^Asp(GTC)^ mediated by RNA methyltransferase activities is critical for stress tolerance in both Arabidopsis and *P. patens* thus indicating this to be an evolutionarily conserved mechanism in land plants.

## Data Availability Statement

The datasets presented in this study can be found in online repositories. NCBI SRA BioProject ID: PRJNA514203.

## Author Contributions

DS, RY, and NW conducted wet lab work, analyzed transcriptome data, and wrote the first draft of the manuscript. SKau analyzed the interactome data. MK and SKap planned the experiments. MK acquired financial support and finalized the manuscript with RY, DS, NW, SKau, and SKap. All authors contributed to the article and approved the submitted version.

## Funding

This work is supported by funds received from the Science and Engineering Research Board (SERB; EMR/2016/000513) and the Department of Biotechnology Government of India (DBT; BT/PR20801/BPA/118/199/2016) to MK.

## Conflict of Interest

SKau was employed by the company Vproteomics, Valerian Chem Private Limited.

The remaining authors declare that the research was conducted in the absence of any commercial or financial relationships that could be construed as a potential conflict of interest.
