# Supplementary figures and images for: Transcriptome Analysis of ppdnmt2 and Identification of Superoxide Dismutase as a Novel Interactor of DNMT2 in the Moss Physcomitrella patens
Source: Front Plant Sci. 2020 Aug 5;11:1185. doi: 10.3389/fpls.2020.01185 (PMC7419982; doi:10.3389/fpls.2020.01185)

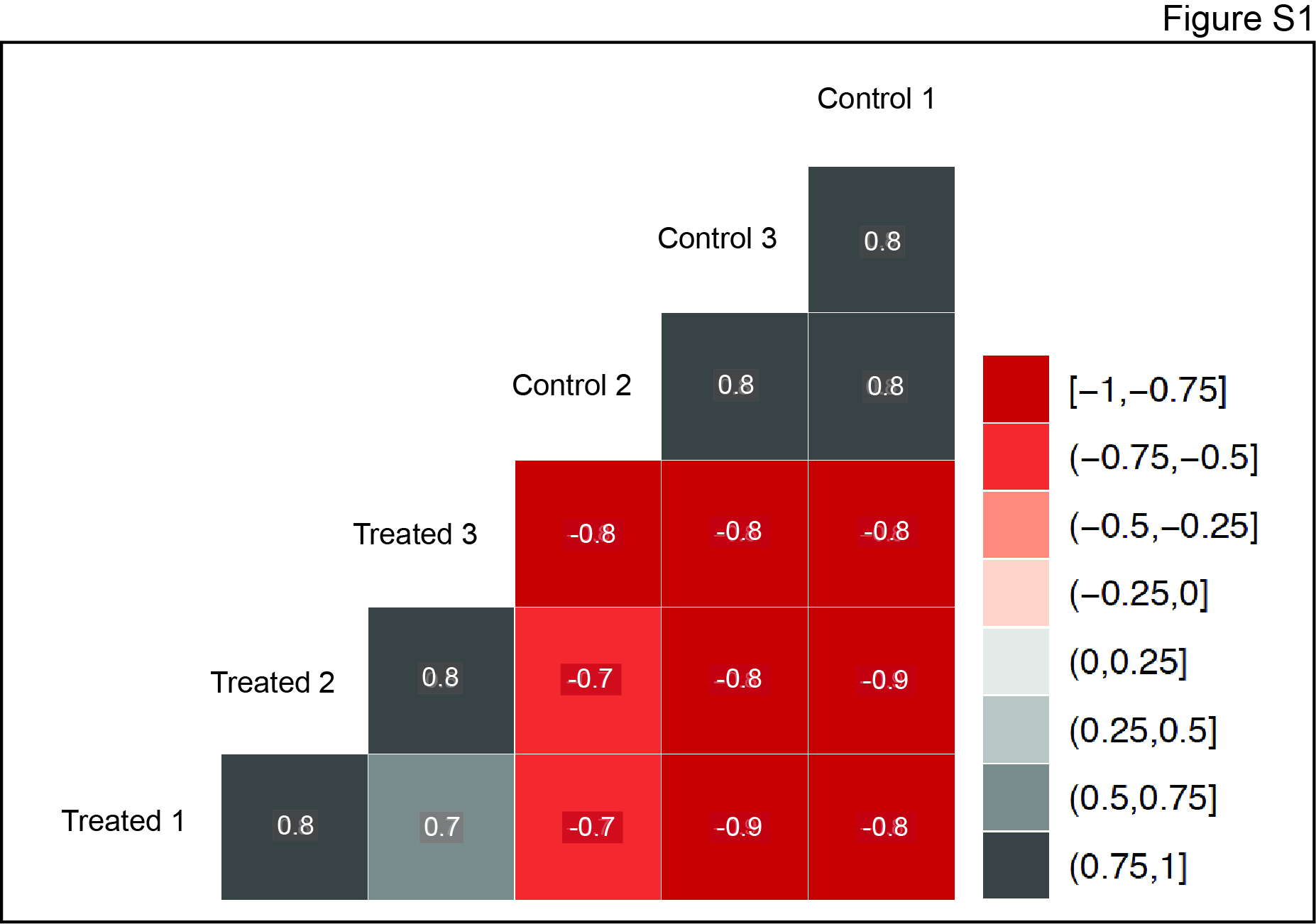

Supplement: Supplementary file 1 [file Image_1.tif]
