# Supplementary material for: Transcriptome Analysis of ppdnmt2 and Identification of Superoxide Dismutase as a Novel Interactor of DNMT2 in the Moss Physcomitrella patens
Source: Front Plant Sci. 2020 Aug 5;11:1185. doi: 10.3389/fpls.2020.01185 (PMC7419982; doi:10.3389/fpls.2020.01185)
Supplement: Supplementary file 2 [file Table_1.pdf]

Table S1: Details of primers used in the study.

For yeast two-hybrid construct preparation	
**Primer Name**	**Primer Sequence (5'-3')**
PpDNMT2TOPOY2HFP	CACCCTTCTCCGTGCTCTTCATCCA
PpDNMT2TOPOY2HRP	CTAGGTGACGAGTTGATTGGGTTCAGA
Pp3c9_25690Y2H_FP	ATAGTCGACATGGCAGCGACGGCGATGGCCA (*Sal*I)
Pp3c9_25690Y2H_FP	ATACTGCAGTTACAGAGGTGTTAGACCCAC (*Pst*I)
